# Application of the qSOFA score to predict mortality in patients with suspected infection in a resource-limited setting in Malawi

**DOI:** 10.1007/s15010-017-1057-5

**Published:** 2017-08-07

**Authors:** Michaëla A. M. Huson, Chawezi Katete, Lilian Chunda, Jonathan Ngoma, Claudia Wallrauch, Tom Heller, Tom van der Poll, Martin P. Grobusch

**Affiliations:** 1 Division of Infectious Diseases, Center of Experimental and Molecular Medicine, Academic Medical Center, University of Amsterdam, Meibergdreef 9, Room G2-105, 1105 AZ Amsterdam, The Netherlands; 2 Division of Infectious Diseases, Center of Tropical Medicine and Travel Medicine, Academic Medical Center, University of Amsterdam, Amsterdam, The Netherlands; 3 Medical Department, Kamuzu Central Hospital, Lilongwe, Malawi; 4 Lighthouse HIV Clinic, Kamuzu Central Hospital, Lilongwe, Malawi

**Keywords:** Sepsis, qSOFA, Resource-limited setting, HIV

## Abstract

**Purpose:**

To determine the predictive value of qSOFA (quick Sequential Organ Failure Assessment) in Malawian patients with suspected infection.

**Methods:**

Prospective observational study in a tertiary referral hospital in Malawi.

**Results:**

Predictive ability of qSOFA was reasonable [AUROC 0.73 (95% CI 0.68–0.78)], increasing to 0.77 (95% CI 0.72–0.82) when classifying all patients with altered mental status as high risk. Adding HIV status as a variable to the qSOFA score did not improve predictive value.

**Conclusion:**

qSOFA is a simple tool that can aid risk stratification in resource-limited settings.

**Electronic supplementary material:**

The online version of this article (doi:10.1007/s15010-017-1057-5) contains supplementary material, which is available to authorized users.

## Introduction

The Third International Consensus Definitions for Sepsis and Septic Shock proposes the use of a simplified SOFA (Sequential Organ Failure Assessment) score, termed quickSOFA (qSOFA), for patients with suspected infection outside intensive care to rapidly identify those at high risk for dying [1, 2]. The qSOFA score includes respiratory rate, altered mental status, and systolic blood pressure which are readily available in any setting. As such, the qSOFA score could be of particular relevance in resource-limited regions. We previously did a retrospective analysis of the qSOFA score in adult patients admitted to hospital in Lambaréné, Gabon, with suspected infection (including bacterial infection and/or malaria). In this setting, a qSOFA score ≥2 had a sensitivity of 87% (95% CI 60–98%) and specificity of 75% (95% CI 70–80%), with an area under the receiver-operating characteristic (AUROC) of 0.83 (95% CI 0.74–0.93) in patients with suspected infection [3]. We report here on the first prospective qSOFA score study in a resource-limited setting. We examined the performance of qSOFA to predict mortality in patients with suspected infection in Kamuzu Central Hospital (KCH), a 750-bed tertiary care hospital in Lilongwe, Malawi, with a catchment population of over 5 million people. A previous study in this hospital demonstrated that altered mental state, which is also included in qSOFA, was associated with high mortality rates [4], but the qSOFA score has not been previously evaluated in KCH.

## Methods

### Data collection

During 6 weeks in November and December 2016, all consecutive patients admitted to the adult medical ward of KCH were included. The qSOFA score was calculated for all with suspected infection, defined by the start of antibiotics, antimalarials or antituberculous medication. The qSOFA score allocates one point for each of the following: respiratory rate ≥22/min, altered mentation [Glasgow Coma Scale (GCS) of ≤14], or systolic blood pressure ≤100 mmHg. Data collection was based on routine clinical observations, supplemented by a dedicated physician who checked all new admissions twice daily and recorded vital signs included in the qSOFA score in case of missing data. Therefore, every patient had a qSOFA score recorded within 12 h of admission. HIV status and final diagnoses were recorded, based on routine clinical observations, as well as in-hospital mortality (the primary study endpoint), and duration of hospital stay. Patients who were transferred out or who absconded were excluded from the analysis. All data were entered into an anonymized excel database. Informed consent need was waived by the ethical board of KCH.

### Statistical analyses

Continuous variables are presented as median with interquartile range [IQR]. Categorical variables are expressed as number and percentage. Comparisons were made using a Mann–Whitney *U* test for continuous variables and Fisher’s exact *t* test for categorical variables. Patients with missing values for the qSOFA score were excluded from the primary analyses. To assess the performances of the qSOFA to predict in-hospital mortality, we calculated sensitivity, specificity, and negative and positive predictive values for a qSOFA score of ≥2. We also calculated the area under the receiver-operating characteristic curve (AUROC). All statistical analyses were 2-tailed, and a *P* value <0.05 was considered significant. GraphPad Prism was used for statistical analyses (GraphPad Software, La Jolla, California).

## Results

During the study period 650 patients were admitted, of which 518 (80%) had a suspected infection. Information on follow-up or qSOFA score was incomplete in 60 patients, leaving 458 patients for analysis. Baseline characteristics are given in Table 1. In-hospital mortality was 23% and 41% of patients had a qSOFA score ≥2, reflecting the very sick population that KCH receives as a tertiary referral center. Mortality was 3% in patients with a qSOFA score of 0, and 13%, 35% and 63% in patients with a qSOFA score of 1, 2, and 3, respectively. A HIV test result was available for 87% of patients, of whom 54% was positive. The most common diagnosis was suspected bacterial infection. The qSOFA score outperformed GCS as a single parameter to predict mortality, with an AUROC of 0.73 (95% CI 0.68–0.78) compared to 0.70 (95% CI 0.63–0.76; Table 2). However, sensitivity was increased from 72% (95% CI 62–80) to 79% (95% CI 70–87) by allocating more weight to the GCS in the qSOFA score, thus classifying both patients with qSOFA ≥ 2 or GCS < 15 as a high-risk group, while specificity decreased only moderately from 68% (95% CI 63–73) to 63% (95% CI 58–68), resulting in an AUROC of 0.77 (95% CI 0.72–0.82).

**Table 1:** Baseline characteristics of study participants

Characteristics	All patients *n* = 458	In-hospital death *n* = 106	Discharged *n* = 352	*P* value
Demographics				
Male, *n* (%)	243 (53.1)	63 (59.4)	180 (51.1)	0.15
Age (years), median [IQR]	35 [26–47]	40 [30–52]	33.5 [25–45]	0.001
HIV status				
HIV status known, *n* (%)	397 (86.7)	92 (86.8)	305 (86.6)	1.0
HIV positive, *n* (%)^a^	213 (53.7)	50 (54.3)	163 (53.4)	0.91
On cART, *n* (%)^b^	166 (77.9)	39 (78)	127 (77.9)	1.0
New HIV diagnosis, *n* (%)^b^	20 (9.4)	4 (8)	16 (9.8)	1.0
qSOFA parameters				
BP systolic (mmHg), median [IQR]	110 [97–122]	110 [91.5–129]	111 [98–121]	0.37
Respiratory rate (breaths/min), median [IQR]	28 [20–32]	28 [24–37.75]	27 [20–32]	0.002
Altered mental status, *n* (%)	117 (25.5)	59 (55.7)	58 (16.5)	<0.0001
qSOFA 0, *n* (%)	69 (15.1)	2 (1.9)	67 (19)	<0.0001
qSOFA 1, *n* (%)	201 (43.9)	28 (26.4)	173 (49.1)	<0.0001
qSOFA 2, *n* (%)	169 (36.9)	64 (60.4)	105 (29.8)	<0.0001
qSOFA 3, *n* (%)	19 (4.1)	12 (11.3)	7 (2)	0.0002
qSOFA ≥ 2, *n* (%)	188 (41)	76 (71.7)	112 (31.8)	<0.0001
Diagnoses				
Suspected bacterial infection^c^	225 (49.1)	63 (59.4)	162 (46)	0.012
Probable bacterial infection^d^	37 (8.1)	5 (4.7)	32 (9.1)	0.22
PTB	30 (6.6)	6 (5.7)	24 (6.8)	0.82
EPTB	40 (8.7)	12 (11.3)	28 (8)	0.33
Cryptococcal meningitis	9 (2)	1 (0.9)	8 (2.3)	0.31
Malaria	35 (7.6)	3 (2.8)	32 (9.1)	0.04
Gastro-enteritis	38 (8.3)	3 (2.8)	35 (9.9)	0.02
cART failure	12 (2.6)	1 (0.9)	11 (3.1)	0.31
Other	14 (3.1)	5 (4.7)	9 (2.6)	0.33
Secondary outcome				
Length of stay (days), median [IQR]	4 [2–7]	2.5 [1–5]	5 [2–7]	<0.0001

*IQR* inter quartile range, *cART* combination antiretroviral therapy, *qSOFA* quick sequential organ failure assessment, *BP* blood pressure, *PTB* pulmonary tuberculosis, *EPTB* extrapulmonary tuberculosis

^a^ Percentage of patients with a known HIV status

^b^ Percentage of HIV positive patients

^c^ Defined by the initiation of antibiotic therapy

^d^ Diagnosis supported by imaging or culture results

**Table 2:** Predictive value of qSOFA

	qSOFA ≥ 2	GCS < 15	qSOFA ≥ 2 and/or GCS < 15	qSOFA + HIV status ≥ 2
Sensitivity, % (95% CI)	72 (62–80)	56 (46–65)	79 (70–87)	87 (78–93)
Specificity, % (95% CI)	68 (63–73)	84 (79–87)	63 (58–68)	44 (38–50)
Positive predictive value, % (95% CI)	40 (33–47)	50 (41–60)	39 (33–46)	32 (26–38)
Negative predictive value, % (95% CI)	89 (84–92)	86 (82–90)	91 (86–94)	92 (86–95)
AUROC (95% CI)	0.73 (0.68–0.78)	0.70 (0.63–0.76)	0.77 (0.72–0.82)	0.68 (0.62–0.74)

*qSOFA* quick sequential organ failure assessment, *GCS* Glasgow coma scale, *AUROC* area under the receiver-operating characteristic

A large patient proportion was HIV positive; so we also assessed the impact of adding HIV status as an additional risk factor to the qSOFA score. By allotting one extra point for HIV seropositivity but still applying a ≥2 cut-off to the total score, sensitivity increased to 87% (78–93) (Table 2). However, specificity decreased to 44% (38–50) and the AUROC fell to 0.68 (0.62–0.74). We also analyzed the performance of the qSOFA score separately in HIV positive and HIV negative patients (Supplementary Table 1 and 2) and found predictive value to be equal in both groups.

## Discussion

We found a reasonably good ability of the qSOFA score to predict mortality with an AUROC of 0.73 (95% CI 0.68–0.78), which could be increased to 0.77 (95% CI 0.72–0.82) when patients with altered mental status were classified as high risk regardless of other qSOFA criteria.

The qSOFA score performance was less in the study reported here compared to our retrospective study in Gabon, where we found an AUROC of 0.83 (95% CI 0.74–0.93). Possibly, the differences are related to severity of illness on presentation, or standards of care. As such, there may not be a one-size-fits-all risk score. qSOFA may serve as a simple tool for risk stratification, but requires further prospective validation in different settings. For example, others have compared qSOFA to the CURB-65 for pneumonia patients and found improved predictive ability of the qSOFA score when age of ≥65 or greater was added as risk factor [5]. The value of such an addition depends highly on the patient population, as the median age of our patients was 35 years, with only 11% of patients aged above 64. We also examined the added value of HIV status in risk stratification; however, in the Malawian setting, this resulted in a very low specificity, leading to misclassification of low-risk patients. Prioritizing limited resources to such a large group of patients is unsustainable, so we do not recommend incorporating HIV status in risk stratification in our setting.

Limitations of our study include the single-center design and lack of a definite diagnosis in most patients. However, this reflects the clinical reality in many resource-limited settings and a risk stratification score should be useful regardless of the final diagnosis at discharge.

In conclusion, we report the first prospective data on the ability of qSOFA to predict mortality in a resource-limited setting and found that the qSOFA score is a simple tool that can aid risk stratification in this environment. However, the predictive performance of qSOFA may vary between patient populations. Our observations were made in a very sick population in a tertiary referral centre, and we emphasize the need to further validate qSOFA in different settings, for example, in primary care in resource-limited areas, where qSOFA might help select patients for referral.

## Electronic supplementary material

Below is the link to the electronic supplementary material.

Supplementary material 1 (PDF 483 kb)
