# Supplementary material for: Application of the qSOFA score to predict mortality in patients with suspected infection in a resource-limited setting in Malawi
Source: Infection. 2017 Aug 7;45(6):893–6. doi: 10.1007/s15010-017-1057-5 (PMC5696439; doi:10.1007/s15010-017-1057-5)
Supplement: Supplementary file 1 — Supplementary material 1 (PDF 483 kb) [file 15010_2017_1057_MOESM1_ESM.pdf]

**Supplementary table 1 Predictive value of qSOFA in HIV positive patients**

| | qSOFA ≥2 | GCS<15 | qSOFA≥2 and/or GCS<15 |
|---|---|---|---|
| Sensitivity, % (95% CI) | 68 (53-80) | 60 (45-73) | 78 (64-88) |
| Specificity, % (95% CI) | 63 (55-70) | 87 (81-92) | 59 (51-67) |
| Positive predictive value, % (95% CI) | 36 (26-46) | 58 (44-72) | 37 (28-47) |
| Negative predictive value, % (95% CI) | 86 (79-92) | 88 (81-92) | 90 (82-95) |
| AUROC (95% CI) | 0.69 (0.61-0.77) | 0.74 (0.65-0.82) | 0.77 (0.69-0.84) |

Abbreviations: qSOFA, quick sequential organ failure assessment; GCS, Glascow coma scale; AUROC, area under the receiver operating characteristic.

**Supplementary table 2 Predictive value of qSOFA in HIV negative patients**

| | qSOFA≥2 | GCS<15 | qSOFA≥2 and/or GCS<15 |
|---|---|---|---|
| Sensitivity, % (95% CI) | 71 (55-84) | 45 (30-61) | 79 (63-90) |
| Specificity, % (95% CI) | 75 (66-81) | 81 (74-87) | 66 (58-74) |
| Positive predictive value, % (95% CI) | 45 (33-58) | 41 (27-57) | 41 (30-52) |
| Negative predictive value, % (95% CI) | 90 (83-94) | 83 (76-89) | 91 (84-96) |
| AUROC (95% CI) | 0.75 (0.67-0.83) | 0.63 (0.53-0.73) | 0.76 (0.67-0.84) |

Abbreviations: qSOFA, quick sequential organ failure assessment; GCS, Glascow coma scale; AUROC, area under the receiver operating characteristic.
